# Challenges and [Possible] Solutions to Optimizing Talent Identification and Development in Sport

**DOI:** 10.3389/fpsyg.2020.00664

**Published:** 2020-04-15

**Authors:** Kevin Till, Joseph Baker

**Affiliations:** ^1^Carnegie Applied Rugby Research (CARR) Centre, Carnegie School of Sport, Leeds Beckett University, Leeds, United Kingdom; ^2^School of Kinesiology and Health Science, York University, Toronto, ON, Canada

**Keywords:** athlete, system, development, holistic, health

## Abstract

The modern-day landscape of Olympic and Professional sport is arguably more competitive than ever. One consequence of this is the increased focus on identifying and developing early athletic talent. In this paper, we highlight key challenges associated with talent (athlete) identification and development and propose possible solutions that could be considered by research and practice. The first challenge focuses on clarifying the purposes of talent identification initiatives such as defining what talent is and how its meaning might evolve over time. Challenge two centers on ways to best identify, select and develop talent, including issues with different approaches to identification, the need to understand the impact of development and the need to have appropriate resourcing in the system to support continued development of knowledge. Finally, we discuss two challenges in relation to the ‘healthiness’ of talent identification and development. The first examines whether a talent identification and development system is ‘healthy’ for athletes while the second focuses on how sport stakeholders could discourage the apparent trend toward early specialization in youth sport settings. Whilst this paper discusses the research in relation to these challenges, we propose multiple possible solutions that researchers and practitioners could consider for optimizing their approach to talent identification and development. In summary, talent is a complex and largely misunderstood phenomenon lacking robust research evidence, and given concerns that it is potentially unhealthy, talent identification and selection at younger ages is not recommended.

## Introduction

The modern-day landscape of Olympic and Professional sport is arguably more competitive than ever. The substantial financial and commercial rewards of winning sporting competitions (e.g., Olympic Gold) or even avoiding relegation (e.g., English Premier League football) mean large resources are invested within national governing bodies and professional sport clubs to achieve success (Till et al., 2019). One approach focuses on identifying and developing early athletic talent into the sporting superstars of tomorrow. This system, commonly known as a Talent Identification and Development System (TIDS; Cobley and Till, 2015; Rongen et al., 2018), has significantly grown within sport over the last 15–20 years and often reflects considerable financial investment. For example, English category 1 soccer academies reportedly invest between £2.3 and £4.9 million per annum (Larkin and Reeves, 2018), while United Kingdom Sport reported spending approximately £100 million per annum on identifying and developing sporting talent (UK Sport, 2015).

Although researchers often dispute the merits of talent as a concept (c.f., Howe, 1998; Baker and Wattie, 2018), the reality of working in sport is that talent identification and selection are often necessary due to limited resources available (e.g., financial, personnel, and facilities). Therefore, a TIDS is an approach to using limited resources in the most efficient way possible. Most sporting organizations and practitioners acknowledge the limitations and consequences associated with the early selection of athletes. However, the resource-limited system requires regular selection across the development pathway according to the sport and context. Therefore, despite the significant financial investment in TIDS, talent identification and development are not straightforward processes. These processes are even more complex with young athletes where numerous physiological, psychological and social factors can impact upon understanding, identifying and developing future athletic talent (Cobley and Till, 2017). Moreover, there are important ethical concerns with the way that talent identification and development are positioned within TIDS (e.g., Bailey and Toms, 2010; Vlahovich et al., 2017; Baker et al., 2018a).

Generally, a TIDS involves five steps in the pursuit of sporting excellence, four that were defined in the early 2000s (Reilly et al., 2000) and one more recent addition. The first four steps include (1) Talent Detection, the discovery of potential performers who are not currently involved in the sport in question; (2) Talent Identification, recognizing participants with the potential at an earlier age to become elite performers in the future; (3) Talent Development, providing athletes with a suitable learning environment to accelerate or realize their potential; (4) Talent Selection, the ongoing process of identifying individuals at various stages of development who demonstrate prerequisite levels of performance – largely involve the traditional approach to talent identification and development. The final step – Talent Transfer, focuses on transfer from one sport to another sport where there are greater opportunities to succeed (MacNamara and Collins, 2015; Rea and Lavallee, 2017). These five steps are common across sporting TIDS and are often operationalized within everyday practice (i.e., identification or selection for the next step of a program is influenced by performance in the previous development environment). TIDS often employ a pyramidal structure whereby at each stage of the system the number of places available decreases and the support provided within the program increases (e.g., higher qualified coaches and increased competition). In order to work optimally, this process requires concurrently integrating talent recruitment (i.e., detection, identification, and selection) and talent development (i.e., proper nurturing of skill acquisition) in the pursuit of future elite performance.

The past few decades have seen a considerable increase in academic reviews summarizing issues related to the identification, selection and development of sporting talent (e.g., Vaeyens et al., 2008; Bailey and Collins, 2013; Baker et al., 2018a, b). This is substantiated by further reviews (e.g., Rees et al., 2016; Johnston et al., 2017; Bergkamp et al., 2019) suggesting the quality of evidence being generated for talent is limited. For example, Johnston et al. (2017) noted that most studies within talent identification focus upon the anthropometric and physical characteristics of athletes with very limited work investigating the cognitive, perceptual and/or psychological factors. More importantly perhaps, very little of this work focuses upon how this research might be applied by those working on the frontlines of TIDS (e.g., TIDS managers, coaches, scouts, and support staff) in terms of optimizing their talent identification and development practices.

In this paper, we highlight three key challenges associated with talent (athlete) identification and development and then propose multiple [possible] solutions that researchers and practitioners could consider for optimizing TIDS according to each challenge. Table 1 summarizes these key challenges and solutions for TIDS practitioners, which are then discussed in the following sections according to the research literature. We feel these challenges have implications for the efficient management of the resources within TIDS and, more importantly, for optimizing opportunities, skill acquisition and health in developing young athletes. Although we have tried to acknowledge the ethical issues in our discussion, the focus is on how to improve the processes of identification and development more generally. Moreover, we have focused upon the key challenges and solutions based on our experiences of researching and working within and outside TIDS collectively over a period of 15–20 years, respectively.

**TABLE 1:** Overview of the challenges and (possible) solutions to Talent Identification and Development.

Challenges		Possible solutions
(1) What are we looking for?	(A) Clarifying definitions – what is talent?	• Understand ‘*what is talent* (it is not a fixed capacity and develops over time) • Talent ID vs. Performance ID – The Matrix • Develop evidence for talent indicators within sport specific systems, including… • Measures and criteria within specific sports that help identify talent • Employ retrospective research/tracking designs to monitor characteristics of young athletes aligned to future success
	(B). Understanding sport and predicting the future	• Develop a performance/mental model for the sport • Design research studies to evaluate sport performance • Develop coaching vision - the ability to predict the future
(2) What are the most effective ways to identify, select, and develop talent?	(A) Identifying Talent	• Consider the timing of talent identification • Allow flexibility to move across (or within a system) – be fluid • Monitor the efficacy of the TIDS decisions • Develop sport specific, multi-disciplinary tools that can monitor athletes reliably over time • Use the Coach as an applied scientist – they have data (although may not know it!). How can this be used and shared?
	(B) Understanding development (biological-psychological-social)	• Coach education – Pediatric Science and Biological-Psychological-Social development • Delay identification or provide more opportunities • Assess maturity status and interpret data according to maturity alongside age • Consider grouping strategies (e.g., shirt ordering and bio-banding) to equalize competition and identification opportunities
	(C) Resourcing the System	• Effective use of resource – creating more opportunities • Supporting coach education and training • Funding basic and applied research
(3) Health considerations for TIDS	(A) Are TIDS appropriate and healthy?	• Awareness of TIDS impact on athlete health • Design appropriate learning and development environments with a balance of activities in and outside of sport • Align day-to-day practitioner behaviors to promote athlete health • Develop TID programs and practices that allow sampling of a range of sports and integrative neuromuscular training
	(B) Is early specialization necessary?	• Clear message that sport sampling is a positive outcome with long-term benefits • Application and reinforcement of the message from key stakeholders, coaches, teachers and parents

## Challenge 1: What Are We Looking For?

### Part A: Clarifying Definitions – What Is Talent?

Talent is a commonly used term in society and can be applied across multiple domains including education, music, and sport. Although commonly used, definitions of talent are inconsistent and unclear, leading to contradictions within both society and science. For example, researchers often talk about talent as an ‘innate ability’ (Baker et al., 2018b) but such terms may have different meanings across different contexts. For instance, talent can be used to describe biological predispositions (e.g., a talent for football), the quality being developed (e.g., nurturing a player’s talent) as well as the players themselves (e.g., football talents). In sport settings, talent has been defined as ‘*the presence or absence of particular skills or qualities identified at earlier time points that correlate to or predict expert future performance’* (Cobley et al., 2012, p3.; Issurin, 2017). Although this definition does not conform to recent calls for clearer definitions of talent (Baker et al., 2018b), it likely captures the key goal of a TIDS – understanding the relationship between current performance (and related variables) and future potential. However, whether this is the way talent is viewed and applied by multiple stakeholders (i.e., coaches, athletes, and administrators) within youth TIDS is questionable. This leaves us with an important question – what does talent look like?

Unfortunately, the existing scientific literature generally has limited high-quality evidence to help practitioners answer the above question and understand how current performance-related variables reflect potential for future performance. For instance, most talent identification research (e.g., Gil et al., 2007; Till et al., 2011; Pion et al., 2015; Woods et al., 2017; Jones et al., 2018) uses cross-sectional research designs at ‘one-off’ time points to assess talent within young athletes. These studies compare a range of characteristics (e.g., anthropometric, physical, psychological, and technical) between playing levels (e.g., school vs. academy; Jones et al., 2018) with the assumption that the differences in characteristics between playing standards equals talent. However, these studies and methodologies only measure performance at that ‘one-off’ specific timepoint with little regard for how such characteristics relate to future performance outcomes (or potential; Johnston et al., 2017). Such an approach assumes that talent is a fixed capacity, which is reflected in performance at that specific timepoint. However, this is highly unlikely considering more recent definitions of talent suggest it is dynamic, emergent, non-optimal and non-linear (De Oliveira et al., 2014; Baker et al., 2018a). As a result, evaluating athlete potential and predicting future adult performance within young athletes remains a central problem for all talent identification researchers and practitioners (Rees et al., 2016).

#### Possible Solutions

**FIGURE 1:**
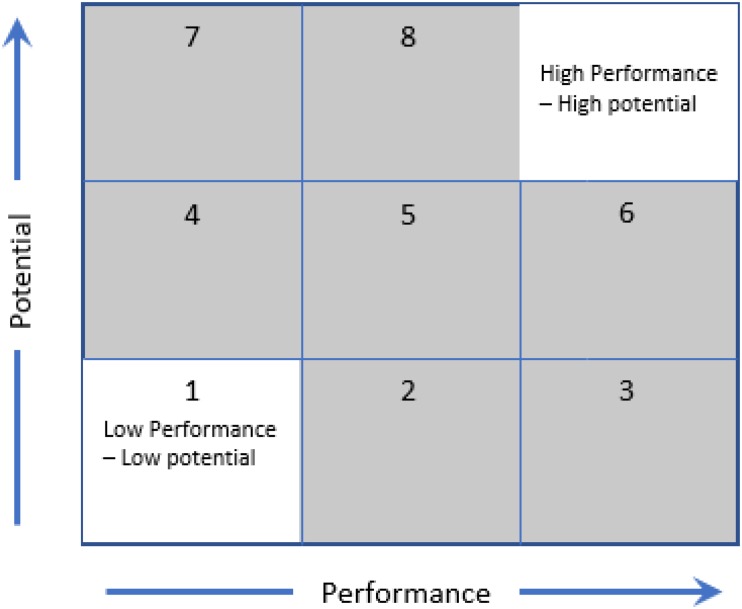
Modified risk matrix from Baker et al. (2018a) highlighting the differing levels of risk when considering athletes for TIDS. Areas with no shading represent ‘low risk’ since performance-based selection systems will remove or keep low versus high performers due to how systems are typically structured (i.e., by confusing potential and current performance). The gray areas represent differing levels of risk that need to be considered relative to resources available in the system (e.g., what is the risk of eliminating a possible 7 through inaccurate selections?).

One solution is for coaches and practitioners to have a clear understanding of “*what talent is*”? and how it relates to their talent identification and development practices. For instance, we recommend positioning talent as (1) emergent [i.e., the process of becoming (Simonton, 1999; Baker et al., 2018b)], (2) influenced by a host of factors within an environment (e.g., parents, coaches, peers, and opportunities; Henriksen et al., 2010; Rees et al., 2016; Davids et al., 2017) and (3) individual (e.g., athletes with different abilities and skills require different developmental programs; De Oliveira et al., 2014). This positioning requires a different approach to talent identification and development than an approach where talent is perceived as a fixed and measurable trait. A critical move forward for sports could include establishing and applying a clear philosophy that values long-term development (i.e., player improvement) over short-term outcomes (e.g., winning and current performance). This approach may have its own unique challenges (e.g., getting ‘buy in’ from stakeholders, managing resources differently) but would be an important step to addressing the balance between what an athlete needs for long-term development and what coaches/teams need for short-term success. Furthermore, practitioners could aim to understand, assess and consider both current performance ability and future potential within their talent identification decisions. The 3 by 3-way matrix of performance vs. potential presented by Baker et al. (2018a) may be a useful tool to start exploring such complexities (see Figure 1). This matrix allows practitioners to consider both athlete current performance (low to high) and future potential (low to high), which may aid talent identification decisions.

In order to make more accurate decisions about athlete identification and selection, practitioners and researchers need to establish which characteristics (talent indicators) are related to potential for future success within sport-specific systems. Recently, researchers have aimed to solve this problem through the implementation of both retrospective and longitudinal research designs (Cobley and Till, 2017; Johnston et al., 2017). These methodologies have compared an athlete’s adolescent performance with their future career-related outcome (e.g., professional vs. non-professional). Such methods help understand what characteristics at an earlier time-point (e.g., adolescence) may contribute toward (un)successful future performance. Ultimately, starting to understand current performance and potential for long-term career related outcomes. Johnston et al. (2017) systematic review (examining soccer, gymnastics, rugby league, Australian football, handball, field hockey, tennis, triathlon, and water polo), demonstrated no clear consensus on which characteristics distinguished between future career outcomes within the respective sports. Arguably, this is due to the sport specific nature of talent identification metrics across the varying ages (i.e., 6–19 years) at initial assessment, the length of time to assessing future career outcome (i.e., 1–10 years), and the wide-ranging testing batteries employed. Interestingly, a range of studies (Le Gall et al., 2010; Ostojic et al., 2014; Till et al., 2011, 2015, 2016) have indicated that advanced size and maturity during adolescence, although influential in the identification process, are ineffective for predicting future career attainment within rugby and soccer. Overall, similar methods used across sports and phases of development would help to better understand the relationship between performance and potential, thereby enhancing practitioners’ talent identification decisions.

### Part B: Understanding Sport and Predicting the Future

As noted above, the main purpose of a TIDS is to identify and develop athletes with the greatest potential for success as adults. As a result, this entails an element of predicting the future. Practitioners must make decisions about individuals based on their predictions of those individual’s future performance capabilities within their sport, alongside how the sport will evolve over time. Therefore, two key questions emerge from this view; do we understand the current and future performance demands of the sport? Both questions are important for understanding the talent identification and development process as we need to be able to identify and develop athletes to train and compete within the future versions of their sport. However, this is certainly not an easy task!

Understanding the current demands of sport involves undertaking a performance-needs analysis (McGuigan, 2014). This needs analysis can include the evaluation of the physical, technical, tactical, and psychological requirements of the sport with a multitude of research available to explore these demands (e.g., Cummins et al., 2013; Kempton et al., 2017; Tabben et al., 2019) alongside the expert understanding of coaches. However, the ability to effectively measure and understand the demands of sport can often be difficult due to the complexity of sports performance. Recent research and the development of technology has resulted in innovative methods and analyses to better help understand sports performance. For example, the recent rise in microtechnology devices (e.g., global positioning systems) to evaluate the physical characteristics of match play has exponentially increased over the last decade with advanced analysis techniques (e.g., peak physical characteristics of match-play within specific durations rather than reporting whole match characteristics) now applied (Whitehead et al., 2018). Moreover, the evaluation of other elements of sports performance (e.g., ecological dynamics, Vilar et al., 2012; complex networks, Ramos et al., 2018; hypernetworks of sports performance, Ribeiro et al., 2019) provide evolving and novel approaches for capturing the complexity of sports performance.

Whilst understanding sports performance is a complex challenge, TIDS make decisions at current timepoints for future versions of the sport. This increases the complexity and involves predicting the future of the sport (and whether athletes will be successful). The evolvement and advancement of sport over time, makes this a difficult challenge. For example, research within soccer has demonstrated increases in the volume of high-intensity running distance alongside the frequency and successfulness of technical characteristics completed during the Premier League between 2006 and 2012 (Barnes et al., 2014; Bush et al., 2015). Within rugby, players’ average body mass has increased from 84 to 105 kg between 1955 and 2015 (Hill et al., 2018) while rules and tactics have changed within boxing since 2013 (Davis et al., 2018). These examples demonstrate the numerous changes to the demands of sports, which may have occurred for multiple reasons (e.g., rule changes, advancements in coaching and sport science) but provide evidence that sports evolve over time. Therefore, understanding the current and future evolvements of the sport are key challenges for enhancing TIDS processes.

#### Possible Solutions

Although understanding current and future evolution of sports performance is difficult, there are several strategies that researchers and practitioners may wish to consider. First, sporting organizations might consider establishing a clear performance model [also known as a mental model; (Richards et al., 2012; Tee et al., 2018)] for their respective sport. A performance model allows a ‘vision’ for organizations to understand and communicate the broad long-term physical, technical, tactical, and psychological aims of their sport, TIDS and program. This would demonstrate a well-defined endpoint for what the TIDS is working toward and allow clear communication within organizational structures for relevant stakeholders (e.g., scouts and coaches) within their talent identification and development processes.

Developing this type of performance model would almost certainly involve more complex evaluations of the demands of the sport than have been previously conducted. For instance, most sporting systems still consider key performance indicators along the dimensions of physical/physiological capabilities, technical and tactical skills, and psychological strategies without much discussion of the reality that there are high levels of interaction between elements within a dimension (e.g., personal beliefs about enjoyment and challenge interact to affect motivation) and between dimensions (e.g., the potential for accurate perceptual-cognitive performance during periods of fatigue is affected by physiological fitness; Schapschröer et al., 2016). However, very few studies have explored how these outcomes interact with each other and, as a result, our understanding of performance indicators remains largely superficial and incomplete.

A solid, evidence-based profile of current performance would help coaches predict how their sport might change over time. However, we also recommend that coaches look to not only anticipate future changes to the sport but to plan to create the future. This recommendation would see coaches not as passive agents within the system who react to changes that are forced upon them, but as proactively engaged in creating the change. Recent research in serial winning coaches (Lara-Bercial and Mallett, 2016) identified vision (alongside philosophy, environment, and people) as a common theme for coaches success. Therefore, coaches may need to carefully consider and articulate their future vision of the sport and feed this into their performance model to establish a future thinking philosophy for identifying and developing talent. This would anticipate how their sport will change in the future as well as how they will drive that change. The speed at which a coach and athlete can adapt to changes may be an important predictor of success.

## Challenge 2: What Is the Most Effective Way to Identify, Select and Develop Sporting Talent?

Across sports, multiple TIDS exist with no current consensus as to the best approach. Two factors that are central to the talent identification process are the timing (i.e., age) when identification occurs and the number of opportunities (i.e., places within a program) available within a given TIDS. For example, consider the differences between two team sports (i.e., soccer, Noon et al., 2015; and rugby union, Till et al., 2020) in the United Kingdom. Soccer selects approximately 15 players for a professional club’s academy from the age of 7–8 years whilst rugby union identifies approximately 120 players at 14–15 years for a Regional Academy program. However, the development programs within these TIDS also differ, ranging from 3 to 4 training sessions and 1 competition per week within soccer, to 1 monthly session and 2–3 annual competition opportunities within rugby. These different organizational and sport-specific TIDS affect the approaches to talent identification, and have implications for the accuracy of selections, impact on player retention, and other outcomes (e.g., resourcing a TIDS and philosophies). In the sections below, we highlight several issues that influence the effectiveness of talent identification and development initiatives.

### Part A: Identifying Talent

Organizations’ talent identification decisions are often informed by recommendations (e.g., from coaches and teachers), and/or subjective (e.g., training/competition observations) and objective (e.g., fitness tests) assessments conducted within youth annual age groups (i.e., Under 15 s; Schorer et al., 2017; Till et al., 2019). Furthermore, the personnel involved in talent identification can range from sport scientists implementing objective assessments, to scouts and coaches watching competition providing subjective evaluations of potential and performance. The multidisciplinary team responsible for identifying talent has a challenging task, especially when organizations do not have a clear understanding and philosophy of ‘what is talent’ alongside a clear performance model (as discussed in challenge #1).

Unfortunately, practitioners may not find the answers they need in the scientific literature. Alongside the cross-sectional methodologies employed within many studies, research has also predominantly focused upon unidimensional measures (e.g., fitness qualities) to predict selection. There have been several recommendations for more multi-disciplinary studies (e.g., Johnston et al., 2017; Mann et al., 2017) and although such studies are available (e.g., Elferink-Gemser et al., 2007; Forsman et al., 2016; Woods et al., 2016), they are rare, especially those using longitudinal and retrospective research designs (as described in challenge #1A). Whilst these studies provide multi-dimensional measures of talent, the utilization of practitioners’ subjective evaluations has been limited. Based on recent studies (e.g., Schorer et al., 2017; Towlson et al., 2019) this work is emerging, but lacks longitudinal designs. To be fair, applying multidisciplinary research designs are challenging and collecting appropriate information on the complex psycho-social factors, technical skills and tactical knowledge involved with sport performance across development is a challenge for all involved in talent identification.

The goal of a talent identification decision is to correctly identify a developing athlete with the potential to become a successful elite performer in their respective sport. However, research on the effectiveness of talent identification decisions is also generally limited (Baker et al., 2018b). The current evidence evaluating talent identification and selection accuracy suggests poor validity (Koz et al., 2012), which decreases further when conducted at younger ages (Güllich, 2014; Till et al., 2016). Therefore, TIDS processes implemented at younger ages (e.g., 7 years in soccer) have been strongly questioned and criticized due to their potential lack of accuracy. Such evidence to date, questions both the early implementation of talent identification alongside the data available to inform such decisions.

#### Possible Solutions

First, organizations might ask themselves two questions regarding their talent identification processes, (1) when should talent identification commence? and (2) why (i.e., what is the reason behind trying to identify talent at this point)? Answering such questions would allow practitioners and their TIDS to understand whether an early talent identification program is appropriate and necessary (Baker et al., 2018a). Whilst answering these questions may still result in an early identification approach, it is then recommended that practitioners and organizations, (1) implement a TIDS that allows athletes to enter and exit (ideally in a seamless fashion) at multiple timepoints within a pathway, and (2) evaluate the long-term (and not so long-term) accuracy of their talent identification decisions. Without an understanding of how accurate coaches and scouts are currently, there is no way of (a) evaluating return on investment (i.e., is early TID a worthwhile initiative) or (b) measuring improvement over time (e.g., using different models, emerging technologies).

Second, sporting organizations might develop multi-disciplinary (i.e., physical, technical, tactical, psychological, and social) objective and subjective talent identification tools that can be used to monitor athlete performance and development over time (see also Cobley and Till, 2017). Such tools would be informed by a clear performance model that understands the attributes required for successful athlete development and developed by a range of stakeholders within an organization (e.g., from scouts to sport scientists). The view that the coach can act as an applied scientist is key here as, although coaches may not know it, they are a rich source of data. Therefore, the development of a system that uses, records, monitors and evaluates a multitude of data types may be key to informing effective talent identification decisions. The recent argument of using actuarial-type judgments (i.e., multidisciplinary explicit decision rules; Den Hartigh et al., 2018), could be useful for both for designing studies and enhancing talent identification processes.

### Part B: Understanding Development (Biological-Psychological-Social)

While talent identification is a difficult and often inaccurate process, a further challenge is that most sports implement talent identification processes within cohorts of young athletes. This process requires decisions about future adult performance being made on youths, whom are influenced by a range of biological, psychological, and social developmental factors. This complicates the talent identification decision-making process considerably. For example, from a biological perspective, growth and maturation are key factors and are generally well understood; maturation reflects the timing and tempo of progress toward the mature adult state and varies considerably during adolescence with differences between boys and girls (Malina et al., 2004a). The growth spurt typically occurs at 12 years in girls (range from 10 to 14 years) and 14 years in boys (range from 12 to 16 years), meaning an understanding of maturity is critical within talent identification due to the strong relationships of maturity with physical performance indicators including size, strength, power, and speed (Malina et al., 2004b; Till and Jones, 2015; Howard et al., 2016).

Unfortunately, other developmental processes are not as well understood, at least as they relate to sport contexts. When we combine the biological-psychological-social development of youths with the policy structures of youth sport (i.e., annual-age grouping), there are multiple implications and challenges for talent identification. For example, the two most common problems highlighted within youth athlete TIDS are; (1) Relative Age Effects and (2) Maturity Selection biases. Therefore, young athletes can be (dis)advantaged within talent identification and youth sport. For example, relatively older (e.g., Cobley et al., 2009; Smith et al., 2018) and earlier maturing (Sherar et al., 2007; Meylan et al., 2010; Till et al., 2010) athletes have increased selection opportunities into TIDS. Interestingly, although this selection inequality favors relatively older and earlier maturing athletes, research within rugby league (Till et al., 2016), rugby union (McCarthy et al., 2016), ice hockey (Deaner et al., 2013), and soccer (Ostojic et al., 2014) has shown greater attainment at the adult professional level for relatively younger and later maturing individuals. This is aligned to the ‘underdog’ hypothesis (Gibbs et al., 2012). Therefore, understanding biological-psychological-social development of children and young people, alongside the policy structures used within youth sport, is a major challenge relevant to TIDS.

#### Possible Solutions

Biological-psychological-social development influences talent identification decisions and the efficacy of such decisions (Johnston and Baker, 2019). Therefore, without an understanding of pediatric science and the processes of biological-psychological-social development in children and adolescents, coaches and practitioners are unable to make informed decisions in relation to athlete performance and potential (Gonçalves et al., 2012). Whilst pediatric exercise science has a large evidence base, the translation and application of such knowledge within coach and practitioner education programs may be limited (Eisenmann, 2017), although this has increased in recent years (e.g., Football Associations Youth Qualifications; Football Associations [FA], 2018). Whilst increasing knowledge may be one solution, policy decisions at the macro level of the sport system may be a further solution. For example, recommendations for delaying talent identification (i.e., the age of identification) and widening talent development opportunities (e.g., allowing more development opportunities) have been suggested (e.g., Baker et al., 2009; Cobley et al., 2009; Till et al., 2014), and implemented in some sports (e.g., rugby; Till et al., 2020).

Linked to delayed talent identification, further policy recommendations to reduce RAEs within TIDS have been made for the past decade (Cobley et al., 2009) and were recently reviewed (Webdale et al., 2019). These strategies include rotating age group cut-off dates, reduced age groups [i.e., 9 months; (Musch and Grondin, 2001)], coach awareness (Helsen et al., 2012), using corrective adjustments (Romann and Cobley, 2015), bio-banding (Cumming et al., 2017), and shirt age ordering (Mann and Van Ginneken, 2017). However, limited evidence exists for the successful reduction of RAEs within youth sport with the efficacy and feasibility of most strategies largely unexplored. In one exception, Mann and Van Ginneken (2017) investigated whether age-ordered shirt ordering could reduce RAEs in young soccer players. Soccer scouts were allocated into three groups; (1) no age information, (2) players’ birthdates or (3) knowledge that the numbers on the playing shirts corresponded to the relative age of the players, and scouts ranked players based on their potential. The study findings showed that for options 1 and 2, a typical relative age bias was found but interestingly when scouts watched the games knowing the shirt numbers corresponded to the relative age of the players, the relative age bias was removed. This highlights a potential solution for reducing RAEs within youth sport when match-play is used for talent identification purposes, but further research is required across multiple sports and contexts.

A possible solution to understand maturity variability is for practitioners to measure the maturity status of young athletes to inform talent identification. Several methods are available (see Lloyd et al., 2014 for an overview) for implementing maturity assessments, which may be directed by resource and time. Maturity information could enhance the interpretation of athlete ability to better inform the potential vs. performance dichotomy discussed in challenge 1, especially when talent is identified from annual-age categories. Recent recommendations (Cumming et al., 2017; Till et al., 2018) have presented methods for comparing physical data assessments according to maturity status.

A further potential solution that combines the above two solutions (i.e., grouping and measuring maturity) is an alternative grouping strategy called ‘bio-banding’ (Cumming et al., 2017, 2018). Bio-banding groups athletes based upon size or maturity status rather than chronological age. However, such grouping still considers technical and psychological development and allows individuals to be moved up or down maturity groups based on a combination of physical, technical, and psychological variables. Bio-banding can be applied for talent identification alongside competition structure, and strength and conditioning programming. Therefore, either comparing talent identification testing data within bio-banded groups or organizing groups for evaluation within match-play and/or training may enhance talent identification practices. Although such a strategy makes sense, to date this has only been applied within environments where athletes have already been identified as talented (i.e., English Premier League soccer academies) and has limited empirical evidence in relation to its success to date (Cumming et al., 2018). Furthermore, the application of this method within community environments for talent identification may be difficult due to the challenges with collecting, organizing and arranging athletes into bio-banded groups when data may not be readily available or accurate.

### Part C: Efficient Use of Resources in the System

While having a strong evidence base from which to create and operate a TIDS is important, the success (however, this is judged) of a system will be affected by the amount and allocation of resources. We commenced this article by highlighting the financial investment of English category one soccer academies (e.g., £2.3–£4.9 million per year; Larkin and Reeves, 2018) and the £100 million of UK Sport (2015). Whilst some TIDS have substantial resources to develop, deliver and support their programs, this is not a luxury of all TIDS. Interestingly, with the plethora of talent research available and recent summaries (e.g., Baker et al., 2012, 2017), little research has considered how system resourcing influences talent identification and development.

**FIGURE 2:**
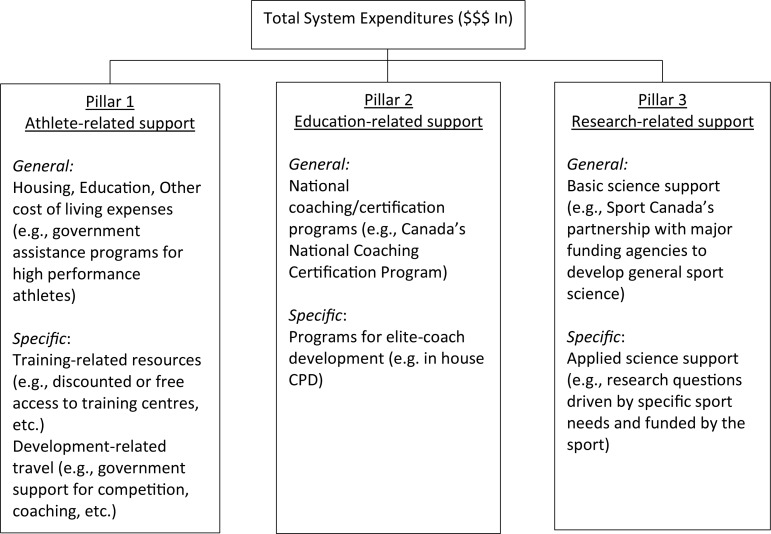
The three pillars of financial resourcing in high performance sport.

On the surface, it would be easy to just assume resourcing reflects total financial resources available to a TIDS. Hogan and Norton’s (2000) examination of financial expenditures in the Australian sport system highlighted the positive relationship between money spent and Olympic medals won, estimating that each medal cost the country approximately $8 million and each gold medal $37 million (see also Johnson and Ali, 2004). Undoubtedly, some level of financial commitment is necessary; however, effective resourcing is more than just gross funding output. In our view, effective management of financial resources integrates three pillars, athlete-related, educated-related and research-related resource and support (see Figure 2).

The system’s most important resource is athlete-related support. Athletes need to be appropriately nurtured in order to maximize the investment made by the TIDS in their development. However, appropriate nurturing assumes we know the factors related to optimal skill acquisition and development in athletes (linked back to challenge #1 and #2). We do not, at least not entirely; nor do we have much understanding of how these factors evolve across the athlete pathway(s). That said, we do know athletes will be required to spend a considerable length of time in intensive practice with high quality coaches in order to acquire the skills necessary for elite performance. From a resourcing perspective, athletes’ engagement in practice is constrained by several factors that can largely be grouped as relating to ‘opportunity.’ For instance, do athletes have the financial support to allow them to devote time to high quality training? Do they have access to the top coaches and competitive peers necessary to further their development? These types of factors relate to opportunities that developing high performance athletes consistently need in order to maximize skill acquisition. In addition to these day-to-day issues, special opportunities arise throughout the development pathway for athletes to experience unique situations (e.g., junior world championships, exposure to traveling teams) that may be important for facilitating the development of key qualities necessary for future elite performance (e.g., development of coping strategies in high pressure settings).

In addition, effective system resourcing also requires attention to factors beyond these immediate (i.e., current athlete) concerns. For instance, effectively educating, training and rewarding coaches to be able to adequately implement the processes of identification and development advocated by each sport is required (Pillar 2). Because the knowledge base is constantly being updated due to advances in research, technology and practice (as discussed earlier), the need for highly trained and knowledgeable practitioners is paramount. Unfortunately, the integration of coaching, training and continuing education programs are not normally considered part of high-performance athlete funding strategies, resulting in a system with short-term focus. To add to this argument, many systems employ a system of progression and incentives that involves coaches advancing from younger to older groups rather become specialized in specific coaching domains. Such factors may be related to resources available for the program and therefore coach education, resource and progression are key factors to consider within the developmental program.

The final pillar relates to resources applied to generating knowledge and evidence to inform the other elements of the system (Pillar 3). On the one hand, this involves providing funding for basic sport science support (i.e., exploratory research instead of agenda-driven research) to identify emergent areas of research that might have value for future coaching and athlete development practice. For instance, research within rugby union academies has tracked the weekly and seasonal workload of youth players using global positioning system technology. Findings highlighted players experience highly variable weekly loads, which may represent concern for optimizing player development alongside negative consequences (e.g., injury, Phibbs et al., 2018a, b). On the other hand, this pillar also relates to resourcing the research priorities of specific high value sports, that may have more immediate needs (e.g., strategies for dealing with extreme heat for the 2022 FIFA World Cup in Qatar). Such examples show the importance of research for enhancing TIDS in the short and long-term.

#### Possible Solutions

The reality of many athlete development systems, particularly those that are government funded, is that financial resources are limited. With this critical constraint in mind, we emphasize the need to improve the efficiency of the overall system so that these limited funds are used more effectively. For instance, knowing that current approaches to talent identification are surprisingly poor (see Baker and Wattie, 2018), a central question relates to how we can support more athletes, for longer, along the pathway. One solution would be a shift away from early identification (as discussed earlier) to longer periods of engagement within the athlete development system, although this would require a different approach to the management of resources and potentially places greater emphasis on understanding athlete needs across the pathway. What is clear from prior work in this area (e.g., Baker and Côté, 2006) is that these needs are not stable and a greater understanding of how, and why, these elements change over time would promote better targeting of resources. Unfortunately, as we have noted earlier, our understanding of the processes of talent development is rather limited and it is difficult to provide clear, evidence-based solutions for athlete resourcing beyond *athletes should have as few barriers to their training as possible*.

Further, management of coach training (Pillar 2) may need to be modified to maximize the limited resources available in this area. In the era of social media, near-constant connectivity and access to big data, traditional approaches to coach education may need to be updated. These emerging platforms emphasize the value of developing stronger communities of practice for coaches and could facilitate sharing information across contexts. A weakness of the current use of these technologies is that it is difficult to separate the ‘signal’ from the ‘noise’ [i.e., the important information from the useless data; (Silver, 2012)]. In the current social media climate where the loudest voice, not the most accurate one, is often the only one that is heard, *high performance sport stakeholders might take a more active role in ‘filtering’ information to their end-users to increase ease of access to high quality information* (e.g., by operating their own social media platforms that deliver high quality information).

Ultimately, effective long-term management of Pillars 1 and 2 would require greater knowledge and evidence delivered in the most effective way to those working at the frontlines of athlete development. One way to provide this support is to encourage greater evaluation of program effectiveness and efficiency (e.g., in developing performance without compromising health) of TIDS by national sport governing bodies and professional clubs including questions about the effectiveness of the program. This type of honest and ‘blame free’ discussion is rare but potentially invaluable. Long-term and consistent improvement only comes from a position of knowing what is working and why.

## Challenge 3: Health Considerations for TIDS

In addition to the key challenges we have noted above, current research and practical insights in this area have highlighted health considerations for athletes involved within TIDS.

### Part A: Are TIDS Appropriate and Healthy?

Talent Identification and Development System have been questioned for their appropriateness and healthiness in the academic literature (Baxter-Jones and Helms, 1996; Lang, 2010; Rongen et al., 2014, 2018) and popular media (especially within soccer; Calvin, 2017; Conn, 2017). Due to the limited effectiveness of a TIDS (discussed in challenge 2A) and that ultimately only a few can make it as a professional athlete, consideration is required for the investment a young athlete gives toward TIDS involvement. This is often above and beyond the time and effort involved in recreational sport, although the time involvement does vary by sport and TIDS. Therefore, potential issues with the appropriateness and healthiness of a TIDS are associated with early specialization (more in 3B; Malina, 2010), increasing the volume and intensity of training (Gonçalves et al., 2012), prioritization of sports (Diehl et al., 2012) and distinct cultures of eliteness (Christensen and Sørensen, 2009).

The commonalities of TIDS, have potential positive and negative outcomes, which have been presented in recent position and consensus statements for youth athletes (Bergeron et al., 2015; Lloyd et al., 2016). Recent communications have presented the fine balance between maximizing positive and negating negative impacts of TIDS involvement (Rongen et al., 2018). These potential impacts include physical (e.g., enhanced physiological capacity vs. injury), psycho-social (e.g., increased confidence vs. development of athletic identity) and educational (e.g., academic high achievers vs. educational sacrifice) outcomes. However, the research evidence to substantiate these impacts is generally limited. For example, recent studies (Read et al., 2018; Tears et al., 2018) have shown contradictory evidence in the injury rates of youth soccer players aged 13–15 years since the commencement of the Elite Player Performance Plan. Although concerns have been raised for TIDS with some evidence available, a well-developed and implemented TIDS can make positive contributions to the health and well-being of youth athletes (Beckmann, 2006; Rongen et al., 2014).

#### Possible Solutions

Whilst TIDS may offer a range of positive and negative health impacts, the potential negative consequences do not lie with the overall concept of talent identification and development (Rongen et al., 2018). Instead these potential negatives reflect how well a TIDS is designed, implemented and managed so that youth athletes can secure positive health outcomes. This is controllable by the practitioners in managing and implementing such programs on a day to day basis. Therefore, a possible solution is for practitioners to be aware that they can promote (positive) and negate (negative) health outcomes through the design of an appropriate learning environment that simultaneously balances multiple training (e.g., load), psychological (e.g., identity), and social (e.g., sense of community) factors that can be challenging for youth athletes (Martindale et al., 2007; Rongen et al., 2014; Bergeron et al., 2015). This environment needs to be established by clear values, expectations and day-to-day routines within the organization, which is a responsibility of all staff connected and engaged with athletes within a TIDS. Placing and communicating athlete health and well-being at the center of a TIDS values, provides opportunities for all practitioners to align their behaviors to promote athlete health through their day-to-day practices.

Alongside the above strategies, numerous training and monitoring solutions can be offered to promote healthy outcomes. For example, fostering an environment that encourages the sampling of a range of sports is recommended (Bergeron et al., 2015; Lloyd et al., 2016). The implementation of integrative neuromuscular training programs (Lloyd et al., 2016) would enable young athletes to develop multiple motor skills and physical qualities needed to transfer sports at a later date, while reducing the injury and psycho-social risks associated with early specialization. Further, the impact of TIDS outcomes could be monitored within practice and over short and long-term outcomes (i.e., what happens in the future). Practitioners could aim to design and implement monitoring and evaluation tools that assess the holistic development of athletes within their TIDS. Such monitoring tools could include a range of factors including athlete wellbeing (Saw et al., 2015; Sawczuk et al., 2017), training load (Phibbs et al., 2017), physical development (Till et al., 2017), injury prevalence (Read et al., 2018) alongside psycho-social factors [e.g., athletic identity (Mitchell et al., 2014); education (Rongen et al., 2014)] and long-term health and performance development (Rongen et al., 2018).

### Part B: How Do We Discourage Early Specialization?

Whilst evidence may be limited on the healthiness of TIDS, early talent identification programs at young ages (i.e., 6–9 years in gymnastics, 8 years in soccer) may promote early specialization (i.e., engagement in intensive year-round training within a single sport; Difiori et al., 2014) within young athletes. Although some sports (e.g., gymnastics, diving, and figure skating) seem to require an early specialization approach due to the early age of peak performance (as early as mid- to late-adolescence), sports often regarded as late specialization sports (e.g., team sports) regularly implement early talent identification processes to increase sport specific practice time whilst competing against other sports for talent (Baker et al., 2018a). At the same time that emerging evidence suggests greater proportions of young athletes are specializing in a single sport earlier in their development, most long-term athletic development models [e.g., Developmental Model of Sports Participation, Côté and Vierimaa, 2014; Long-Term Athlete Development (LTAD) model, Balyi and Hamilton, 2004; Australia’s Foundations, Talent, Elite, Mastery Model; Gulbin et al., 2013] have moved to emphasize the importance of ‘sampling’ a range of sports during youth.

The benefits of this approach to talent identification has been hotly debated compared to a diversified approach to sports participation (Moesch et al., 2011; Fransen et al., 2012). Although early specialization may enhance sport-specific performance in the short-term (i.e., technical skills, decision-making; Ford et al., 2012), this approach may result in the negative health outcomes mentioned above (e.g., injury, overtraining, and burnout) in challenge 3A. Therefore, although early talent identification may have some benefits to sports performance, it is again questionable whether such programs are potentially appropriate and healthy for young athletes. Perhaps equally important for those working in high performance athlete development environments, there seems to be little evidence that early specialization is necessary for future long-term success (e.g., Baker, 2003; Baker and Côté, 2006). Given the risks of early specialization and the lack of evidence for its value in long-term athlete development, advocating for this approach is clearly unwarranted. That said, the pressure on young athletes to specialize in their sports as early as possible is difficult to overcome.

#### Possible Solutions

While the messaging against specialization is clear, the trend continues in many youth sports. One solution would be having clear messages for all stakeholders in the sport system regarding the importance of a broad base of sport experience for the development of elite skill. While policy makers seem to be aligned, parent and coach views are inconsistent. This approach (i.e., greater variability in experience leading to greater skill development) seems counter-intuitive to many parents and coaches so greater emphasis needs to be placed on the mechanisms explaining this relationship (i.e., *why* sampling improves skill development). In addition, the definitions of ‘early specialization’ have been inconsistent and, as a result, we have little understanding of why early specialization is problematic (i.e., what is the mechanism driving these negative effects?).

Focusing on the mechanism(s) could be important for improving messaging and policy. For instance, presenting the importance of sport sampling from an ‘assets building approach’ (i.e., by participating in a broad range of sports you gain a broad base of skills that can make you a better all-round athlete and more resilient to injury) may be beneficial. Such an approach may be more effective in making the case for diversification compared to the typical approach which focuses on ‘risk reduction’ (i.e., do not specialize because it increases your likelihood of getting injured). Ultimately, young athletes’ ability to partake in a broad range of sports may be limited by external factors including messages from their coaches (do coaches pay more than ‘lip service’ to the value of sport sampling?), reinforcement from their parents (what messages do they get from parents?) and opportunities within the system (does the system limit opportunities due to cost, location, etc?). It is also important to acknowledge that the relationships between early specialized training, skill acquisition and health outcomes is much more nuanced than prior work suggests.

## Conclusion

This paper aimed to highlight the key challenges associated with talent identification and development and propose multiple [possible] solutions that researchers and practitioners could consider for optimizing TIDS. The challenges included (1) Understanding what we are looking for (i.e., what is talent; understanding sport performance and predicting the future), (2) Determining the most effective ways to identify, select and develop talent (i.e., identifying talent; understanding biological-psychological-social development; resourcing the system) and (3) Understanding the health considerations of TIDS (are TIDS healthy?; is early specialisztion necessary).

To overcome these challenges, we proposed multiple [possible solutions] to each challenge. Whilst the research evidence base is less established to support these solutions, we hope that these provide considerations for practitioners (policy makers to coaches) and researchers to consider when implementing talent identification and development. Policy makers within sport must consider the evidence base for their TIDS to establish ethical and effective systems. This can include the timing and opportunities available within TIDS, the structure of youth sport, coach education and other resource related factors (e.g., research). Sporting organization professionals and coaches should consider their understanding of talent and athlete development (biological, psychological, and social), develop clear performance models through a thorough (current and future) understanding of their sport, and deliver and monitor programs that focus on athlete health and wellbeing alongside sporting performance. Finally, researchers need to conduct multi-dimensional and longitudinal studies that consider the effectiveness of TIDS to help practitioners and policy makers have a clear understanding of what talent is and how it can be developed.

In summary, our recommendation is that because it is a complex and misunderstood phenomenon, lacking robust research evidence, difficult to assess and potentially unhealthy, we should stop thinking about talent *per se* (especially at younger ages). It may be more effective, and ethical, to apply appropriate and research informed practices to everyone (or as many as possible) for as long as possible. Such an approach may result in greater utilization of resources whilst having the potential to improve both performance and health for everyone in the long-term resulting in a more efficacious system. Whilst we have aimed to articulate the challenges and solutions for TIDS, we acknowledge that this article is based upon the experiences of only two academics researching and working within TIDS over the last two decades. We hope that the article provides stimuli for advanced debate, future work and reflections from all involved in the identification and development of sporting talent.

## Author Contributions

KT and JB conceptualized the idea, wrote and checked the review manuscript.

## Conflict of Interest

The authors declare that the research was conducted in the absence of any commercial or financial relationships that could be construed as a potential conflict of interest.
